# Isolation and characterization of an IGROV-1 human ovarian cancer cell line made resistant to Ecteinascidin-743 (ET-743)

**DOI:** 10.1054/bjoc.2000.1224

**Published:** 2000-04-27

**Authors:** E Erba, D Bergamaschi, L Bassano, S Ronzoni, G Di Liberti, I Muradore, S Vignati, G Faircloth, J Jimeno, M D'Incalci

**Affiliations:** Cancer Pharmacology Laboratory, Department of Oncology, Istituto di Ricerche Farmacologiche ‘Mario Negri’, via Eritrea, Milan, 62-20157, Italy; PharmaMar USA Inc., 26 Landsdowne Street, Cambridge, MA, USA; PharmaMar SA Research and Development, c/Caldera 3, 28760 Tres Cantos, Madrid, Spain

**Keywords:** natural compound, Ecteinascidin-743, drug-resistance, P-gp expression

## Abstract

By exposing Igrov-1 human ovarian cancer cells to increasing concentrations of Ecteinascidin-743 (ET-743), either for a short or prolonged time, we obtained sublines resistant to ET-743 which overexpress Pgp. The most resistant clone (Igrov-1/25 ET) was evaluated for biological and pharmacological characterizations. The increased Pgp levels of Igrov-1/25 ET were not due to amplification of the mdr-1 gene but to increased mRNA levels. No increase in other multidrug resistance-related proteins such as MRP or LRP was observed in Igrov-1/25 ET. The IC_50_values of ET-743 against Igrov-1/25 ET was approximately 50 times higher than the parental cell line. Resistance was not reversed while maintaining the cell line in drug-free medium for at least 24 months. Igrov-1/25 ET was cross-resistant to Doxorubicin and VP16 while it was equally sensitive to L-PAM, MNNG, CPT and only marginally less sensitive to Cis-DDP and Oxaliplatin compared to the parental cell line. Igrov-1/25 ET exposed to Doxorubicin retained this drug much less, mainly because of a more efficient drug efflux. The cyclosporine analogue SDZ PSC-833 reversed the resistance of Igrov-1/25 ET to ET-743, without any enhancement of the drug activity against the parental Igrov-1 cell line. Igrov-1/25 ET exhibits typical features of cell lines overexpressing the mdr-1 gene and can be a potentially useful tool in selecting ET-743 non-cross-resistant analogues as well as to investigate methods to counteract resistance to this drug. © 2000 Cancer Research Campaign


British Journal of Cancer (2000) **82**(10), 1732–1739
© 2000 Cancer Research Campaign
DOI: 10.1054/ bjoc.2000.1224, available online at http://www.idealibrary.com on IDEAL®

### Isolation and characterization of an IGROV-1 human ovarian cancer cell line made resistant to Ecteinascidin-743 (ET-743)

**E Erba[1], D Bergamaschi[1], L Bassano[1], S Ronzoni[1], G Di Liberti[1], I Muradore[1], S Vignati[1], G Faircloth[2], J Jimeno[3] and M D'Incalci[1]**

[1]Cancer Pharmacology Laboratory, Department of Oncology, Istituto di Ricerche Farmacologiche 'Mario Negri', via Eritrea 62-20157 Milan, Italy; [2]PharmaMar USA, Inc., 26 Landsdowne Street, Cambridge, MA, USA; [3]PharmaMar SA, Research and Development, c/Caldera, 3, 28760 Tres Cantos, Madrid, Spain

**Summary** By exposing Igrov-1 human ovarian cancer cells to increasing concentrations of Ecteinascidin-743 (ET-743), either for a short or prolonged time, we obtained sublines resistant to ET-743 which overexpress Pgp. The most resistant clone (Igrov-1/25 ET) was evaluated for biological and pharmacological characterizations. The increased Pgp levels of Igrov-1/25 ET were not due to amplification of the mdr-1 gene but to increased mRNA levels. No increase in other multidrug resistance-related proteins such as MRP or LRP was observed in Igrov-1/25 ET. The $IC_{50}$ values of ET-743 against Igrov-1/25 ET was approximately 50 times higher than the parental cell line. Resistance was not reversed while maintaining the cell line in drug-free medium for at least 24 months. Igrov-1/25 ET was cross-resistant to Doxorubicin and VP16 while it was equally sensitive to L-PAM, MNNG, CPT and only marginally less sensitive to Cis-DDP and Oxaliplatin compared to the parental cell line. Igrov-1/25 ET exposed to Doxorubicin retained this drug much less, mainly because of a more efficient drug efflux. The cyclosporine analogue SDZ PSC-833 reversed the resistance of Igrov-1/25 ET to ET-743, without any enhancement of the drug activity against the parental Igrov-1 cell line. Igrov-1/25 ET exhibits typical features of cell lines overexpressing the mdr-1 gene and can be a potentially useful tool in selecting ET-743 non-cross-resistant analogues as well as to investigate methods to counteract resistance to this drug. © 2000 Cancer Research Campaign

**Keywords**: natural compound; Ecteinascidin-743; drug-resistance; P-gp expression

Ecteinascidin-743 (ET-743) is a novel anticancer drug that is produced by the marine tunicate *Ecteinascidia turbinata* (Rinehart et al, 1990; Guan et al, 1993; Jimeno et al, 1996). It is a carbinolamine containing antibiotic composed of three tetra-hydroisoquinoline subunits. According to an NMR-based model, ET-743 interacts with the minor groove of DNA and alkylates guanine at the N2 position (Moore et al, 1997). Alkylations of guanines were also demonstrated by evaluating the changes in electrophoretic mobility of guanine-containing oligonucleotides reacting with ET-743 occurring after washing with a denaturing sodium dodecyl sulphate (SDS)-containing solution to remove drug molecules reversibly bound to DNA (Pommier et al, 1996; Zewail-Foote and Hurley, 1999). The novel structure, unique mechanism of interaction with DNA and the potent preclinical antitumour activity observed against human cancers (Ghielmini et al, 1998; Valoti et al, 1998), understandably makes this drug an attractive candidate for clinical investigation.

The results recently obtained in the phase I clinical trials are very encouraging as some objective responses have been observed for doses of ET-743 associated with manageable toxicity (Cvitkovic et al, 1999; Rayan et al, 1999).

Considering the pharmacological and clinical interest in this compound we have attempted to obtain cell lines resistant to ET-743 by using two different protocols, 1 h exposure or 24 h exposure, of the human ovarian cancer Igrov-1 cell line to better understand the possible mechanism(s) of resistance for this drug. In this paper we report the results of these studies and a detailed characterization of the most resistant cellular clone.

*Received 16 September 1999*
*Revised 13 January 2000*
*Accepted 18 January 2000*

*Correspondence to:* E Erba

### MATERIALS AND METHODS

### Cells and culture conditions

The human ovarian carcinoma Igrov-1 cell line was grown in monolayer in RPMI-1640 medium supplemented with 10% fetal calf serum, 1% glutamine 200 mM (Gibco Europe, Paisley, UK) at 37°C in a humidified 5% carbon dioxile ($CO_2$) atmosphere in T25 cm$^2$ tissue flasks (IWAKI, Bibby Sterilin, Staffordshire, UK) (Bénard et al, 1985).

Igrov-1/ET-743 resistant cells were derived from the Igrov-1 cell line by stepwise selection with ET-743 (kindly supplied by PharmaMar, S.A. Tres Cantos, Spain). The cells were treated for 10 months with increasing concentrations at short (1 h) or long (24–48 h) exposure times, starting from 20 and 0.5 nM respectively. The concentrations were increased by a factor ranging from 1.5 to 2. The maximal ET-743 concentration reached was 800 nM and 70 nM for short and prolonged exposure protocols respectively. The clonal subpopulations were obtained by isolating individual colonies of Igrov-1 subline made resistant to ET-743 by using the short exposure protocol. Briefly, cells were plated at 900 cells per 87-mm tissue culture Petri dish (IWAKI) in 10 ml of culture medium. The cells were grown until individual cells had formed single colonies of approximately 50 cells. The colonies were separately sub-cultured using cloning rings and transferred to a single well of a 96-well plate. When the cells reached 90% confluency they were sub-cultured into a single 48-well plate and subsequently in a 25 $cm^2$ flask. The cell line derived from clone 25 which was the most resistant to ET-743, Igrov-1/25 ET, was cultured in medium in absence of ET-743.

### Clonogenicity test

Resistance to ET-743 was evaluated using a standard clonogenic assay. Exponentially growing Igrov-1 and Igrov-1/25 ET cells were treated for 1 h with different concentrations of ET-743, doxorubicin (dox), melphalan (L-PAM), etoposide (VP-16), cisplatin (cis-DDP), Oxaliplatin, camptotecin (CPT) and *N*-methyl-*N*-nitro-*N*-nitrosoguanosine (MNNG). After treatment, cells were washed twice with phosphate-buffered saline (PBS). Then 3000 control or treated cells were plated in 50-mm tissue culture dishes with 3 ml fresh medium. The colonies were allowed to develop for 10–14 days. Plating efficiency of the exponentially growing control cells was between 40 and 50%. The colonies were stained with 1% crystal violet solution in 20% ethanol, and the number of colonies were measured using the Entry Level image system (Immagini & Computer, Bareggio, Milan, Italy). A background correction was made and the smallest control cell colony was taken as the minimum for setting the cut-off point.

To evaluate the modulation of ET-743 cytotoxicity by the mdr phenotype reversing agent SDZ PSC-833 (PSC), both Igrov-1 and Igrov-1/25 ET cell lines were treated with different concentrations of ET-743 alone or in combination with 1 μM PSC for 1 h. The cytotoxicity was evaluated by the colony previously described.

The analysis of variance, stratified by drug concentrations, was performed to assess the differences in the dose–response curves.

### Flow cytometric DNA, protein and cytokeratin content

DNA, protein and cytokeratin content of Igrov-1 and Igrov-1/25 ET cell line were evaluated by standard flow cytometric methods (Broggini et al, 1988; Ferrero et al, 1990).

### Flow cytometric analysis of multidrug-related proteins

Cells were detached from the tissue culture plates by trypsinization, washed with PBS and then counted. Cells ($10^6$) were washed with PBS and 2% human serum (HS) and incubated for 60 min with 150 μl anti-P-glycoprotein (P-gp) antibody, clone MRK-16, diluted 1:30 in PBS + 2% HS at room temperature in the dark (Kamiya Biochemical, Seattle, WA, USA). Then the cells were washed in PBS + 2% HS and incubated with a fluorescein isothiocyanate (FITC)-conjugated AffiniPure $F(ab')_2$ fragment goat anti-mouse IgG (Jackson Immuno Research Lab. Inc., West Grove, PA, USA) diluted 1:50 for 1 h at room temperature in the dark.

Multidrug resistance-associated protein (MRP) and lung resistance-related protein (LRP) were evaluated in cells after permeabilization in 1% (v/v) lysing solution G (Becton Dickinson, San Jose, CA, USA) in $dH_2O$ and incubated for 10 min in PBS/BSA containing 1% (v/v) normal goat serum (NGS). Cells ($10^6$) were incubated for 1 h at 4°C in 150 μl antibody (anti-MRP and anti-LRP, clone MRPr1 or clone LRP-56 respectively, diluted 1:30 in PBS/BSA containing 1% NGS in the dark (Kamiya Biochemical).

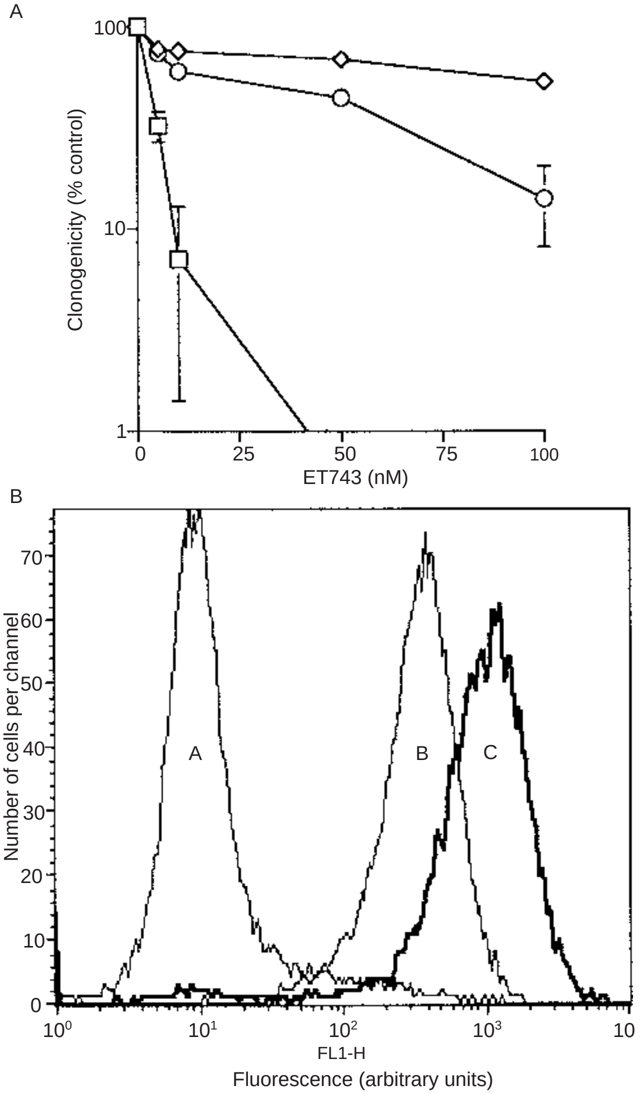

**Figure 1** (**A**) Effects of ET-743 on the clonogenicity of Igrov-1 parental cell line (□) and the two clones made resistant to ET-743 by step-wise increase in drug concentration and by using either short (◇) or prolonged drug exposure protocols (○). Points are the mean of six independent replicates; bars represent s.d. (**B**) Flow cytometric analysis of P-gp expression of Igrov-1 parental cell line (A) and of the two clones made resistant to ET-743 by step-wise increase in drug concentration and by using either short (B) or prolonged drug exposure protocols (C)

Cells were washed in PBS/BSA + 1% NGS and incubated with a FITC-conjugated AffiniPure $F(ab')_2$ fragment goat anti-rat IgG 1:100 for MRP or with a FITC-conjugated AffiniPure $F(ab')_2$ fragment goat anti-mouse IgG 1:50 for LRP (Kamiya Biochemical). Cells were resuspended in PBS and the flow cytometry analyses were performed on at least 20 000 cells for each sample by the FacSort cytometry system (Becton Dickinson) (Krishan et al, 1997). Igrov-1, A2780, LoVo/DX and POGB/DX, kindly supplied by Dr R Supino, Istituto Nazionale dei Tumori, Milan (Binaschi et al, 1995), were used as positive controls.

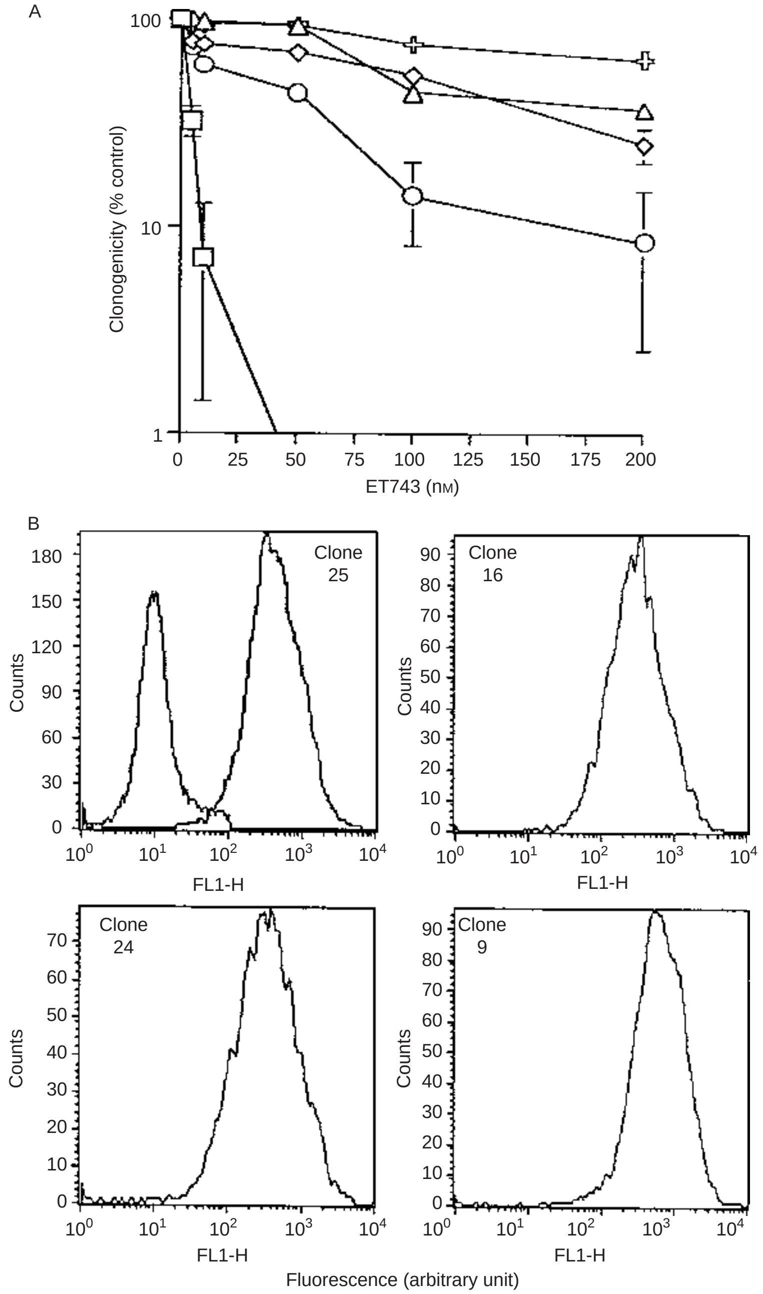

**Figure 2** (**A**) Effects of ET-743 on the clonogenicity of Igrov-1 parental cell line and the four clones obtained from Igrov-1 subline made resistant to ET-743 by using the short drug exposure protocols. Points are the mean of six independent replicates; bars represent s.d. (□) = Igrov-1; (◇) = Clone 9; (○) = Clone 16; (△) = Clone 24; (✣) = Clone 25. (**B**) Flow cytometric analysis of P-gp expression of the four clones obtained from Igrov-1 subline made resistant to ET-743 by using the short drug exposure protocols. In the histogram of Clone 25 the Igrov-1 parental cell line (first histogram on the left) is also shown

### Flow cytometric studies of dox uptake and efflux

Exponentially growing Igrov-1 and Igrov-1/25 ET cells were incubated for 4 h at 37°C in 5% $CO_2$ with different concentrations of dox. Then control and dox-treated cells were centrifugated at 4°C and resuspended in new medium. At different time intervals during dox incubation (uptake) and at 15, 30, 60, 120 and 240 min after drug-washout (efflux), the total fluorescence was measured by Facs Star Plus (Becton Dickinson). Dox fluorescence was detected with excitation at 488 nm and emission above 530 nm. The analyses were performed on at least 50 000 cells and the data are the mean of three independent replicates. Dead cells were excluded on the basis of their scatter signals. Measurements were corrected for the contribution of fluorescence of untreated cells (Limonta et al, 1991).

### DNA blot analysis

Genomic DNA from cells was isolated according to standard procedures and digested with *Eco*RI. Digested DNAs (25 µg) were size fractioned on 0.8% gel agarose and transferred to nylon membrane (Gene Screen Plus, NEN, Boston, MA, USA) as described (Sambrook et al, 1989).

*© 2000 Cancer Research Campaign*

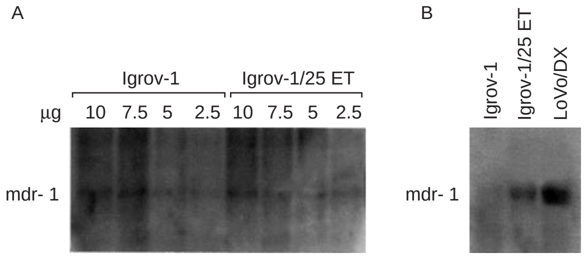

**Figure 3** (**A**) Southern blotting analysis in Igrov-1 and Igrov-1/25 ET cells. Serial dilution of DNA obtained from both cell lines were digested with *Eco*RI and separated on agarose gel. Hybridization with mdr-1 probe and carried out as described in Materials and methods. (**B**) mRNA levels of the mdr-1 gene in Igrov-1 and Igrov-1/25 ET cells. LoVo/DX RNA was loaded as positive control for mdr-1 overexpression

### RNA blot analysis

Total cellular RNA was extracted by the guanidine isothiocyanate/caesium chloride centrifugation (Sambrook et al, 1989). For Northern blot analysis 10 µg of total RNA was fractionated on 1% agarose gel containing 6.7% formaldehyde and transferred to nylon membrane (Gene Screen Plus). LoVo/DX mRNA was used as positive control (Broggini et al, 1988). Levels of gene amplification or mRNA expression were determined by densitometry of the autoradiograms. To normalize the amounts of loaded DNA or RNA, the slot blots were rehybridized with a β-actin probe.

### Hybridization

The filters were pretreated for at least 1 h at 42°C in 50% formamide, 10% dextran sulphate, 1 M sodium chloride, 1% SDS and then hybridized overnight in the same mixture containing 100 mg $ml^{-1}$ of denaturated salmon sperm DNA and $1 \times 10^6$ cpm $ml^{-1}$ of [α- $^{32}P$] d-CTP labelled probes. Labelled probes were obtained using the Rediprime Kit (Amersham Italia S.r.1., Milan, Italy) utilizing the 1.3 Kb *Eco*RI-Sal1 insert of human mdr-1 gene (pc Dr 1.3) and the 1.3 Kb *Pst*1 insert of the murine α-actin gene. After hybridization, the membranes were washed twice at room temperature in 2 × SSC (1 × SSC is 0.15 M sodium chloride–0.015 M sodium citrate) and once at 65°C in 2 × SSC and 1% SDS and then exposed to Kodak XRP film at –80°C with an intensifying screen.

### RESULTS

### Selection and characterization of cells

Figure 1A shows the effect of ET-743 on the clonogenicity of Igrov-1 and of the two sublines made resistant to ET-743 by stepwise increase in drug concentration and by using either prolonged (○) or short drug exposure protocols (◇). Both cell lines expressed the multi-drug resistance protein P-gp assessed by using the monoclonal antibody MRK16 (Figure 1B).

From Igrov-1 subline made resistant by using the short exposure protocol 30 subclones were isolated and all were found to express Pgp.

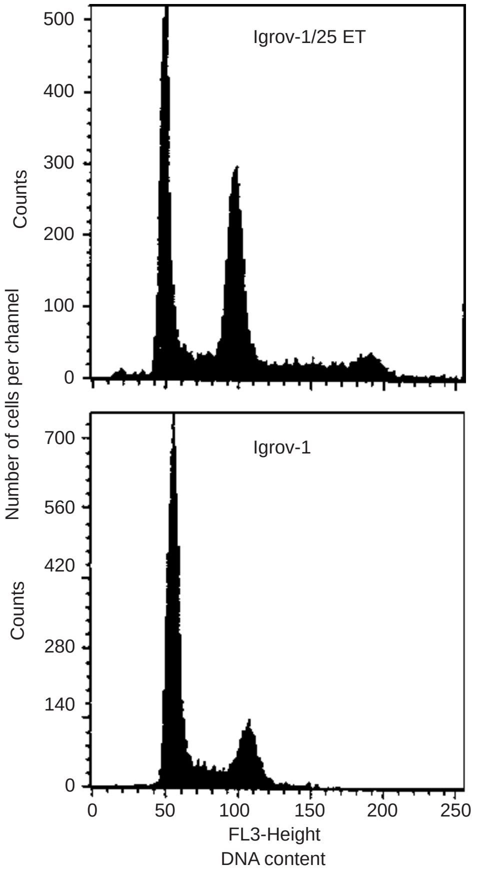

**Figure 4** Flow cytometric DNA content analysis on Igrov-1/25 ET and Igrov-1 parental pcell lines

Out of these 30 clones we selected four clones to investigate the cytotoxicity of ET-743 by clonogenic assay (Figure 2A). The $IC_{50}$ of the four clones varied markedly in spite of the fact that the level of the P-gp expression appeared to be similar in the different clones, being the means fluorescence channel 316, 339, 334 and 454 in the clones 25, 16, 24 and 9 respectively (Figure 2B). We then selected clone 25 (Igrov-1/25 ET) for further characterization because it was the most resistant to ET-743.

In order to evaluate if the increased P-gp expression was due to the amplification of mdr-1 gene and/or to increased mRNA levels Southern and Northern blots analyses were carried out. No amplification of the mdr-1 gene was found (Figure 3A). A threefold increase in the mRNA levels in the Igrov-1/25 ET cell line with respect to the parental cell line was detected by densitometric analysis (Figure 3B). These data indicate that the increased expression of P-gp was due to an increase in the level of mRNA of mdr-1 gene and not to amplification of mdr-1 gene.

Morphology and growth characteristics of Igrov-1/25 ET cell line were consistent with the parental Igrov-1 cell line. Igrov-1 and Igrov-1/25 ET cell lines did not differ with respect to doubling times, about 23 h for both, immunostaining of total protein or cytokeratin content. Flow cytometric analysis showed that in both Igrov-1 and Igrov-1/25 ET cell lines ET-743 caused a dramatic block in $G_2$ phase that was evident at concentrations of 20 and 200 nM respectively. DNA content analysis showed in Igrov-1/25 ET cell line the appearance of a new cell population containing

*© 2000 Cancer Research Campaign*

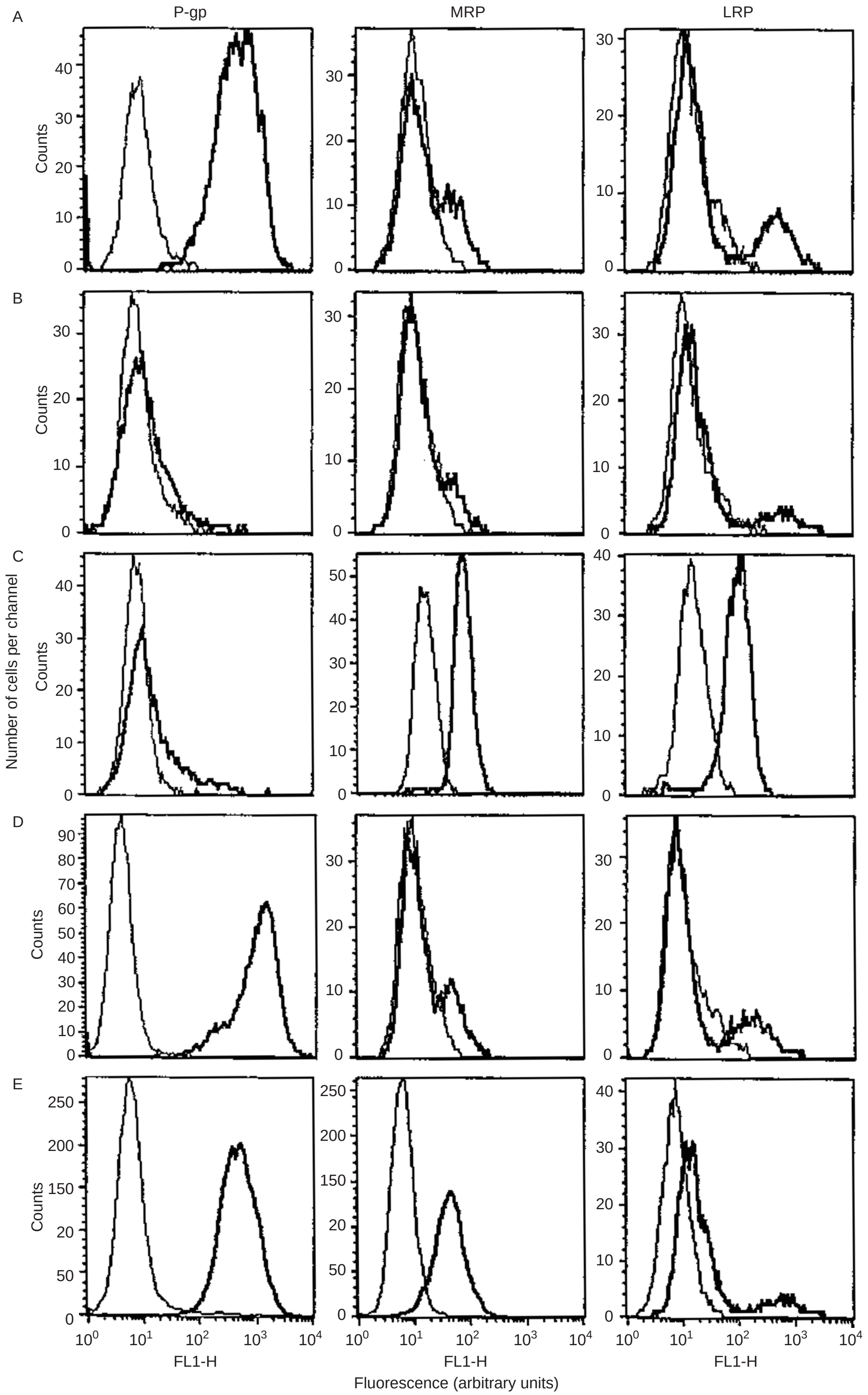

**Figure 5** Flow cytometric analysis of P-gp, MRP and LRP expression in Igrov-1/25 ET (**A**), Igrov-1 (**B**), A2780 (**C**), LoVo/DX (**D**) and POGB/DX (**E**). Thin black line: negative control, w/o addition of specific antibody; bold black line: cell line with addition of specific antibody

*© 2000 Cancer Research Campaign*

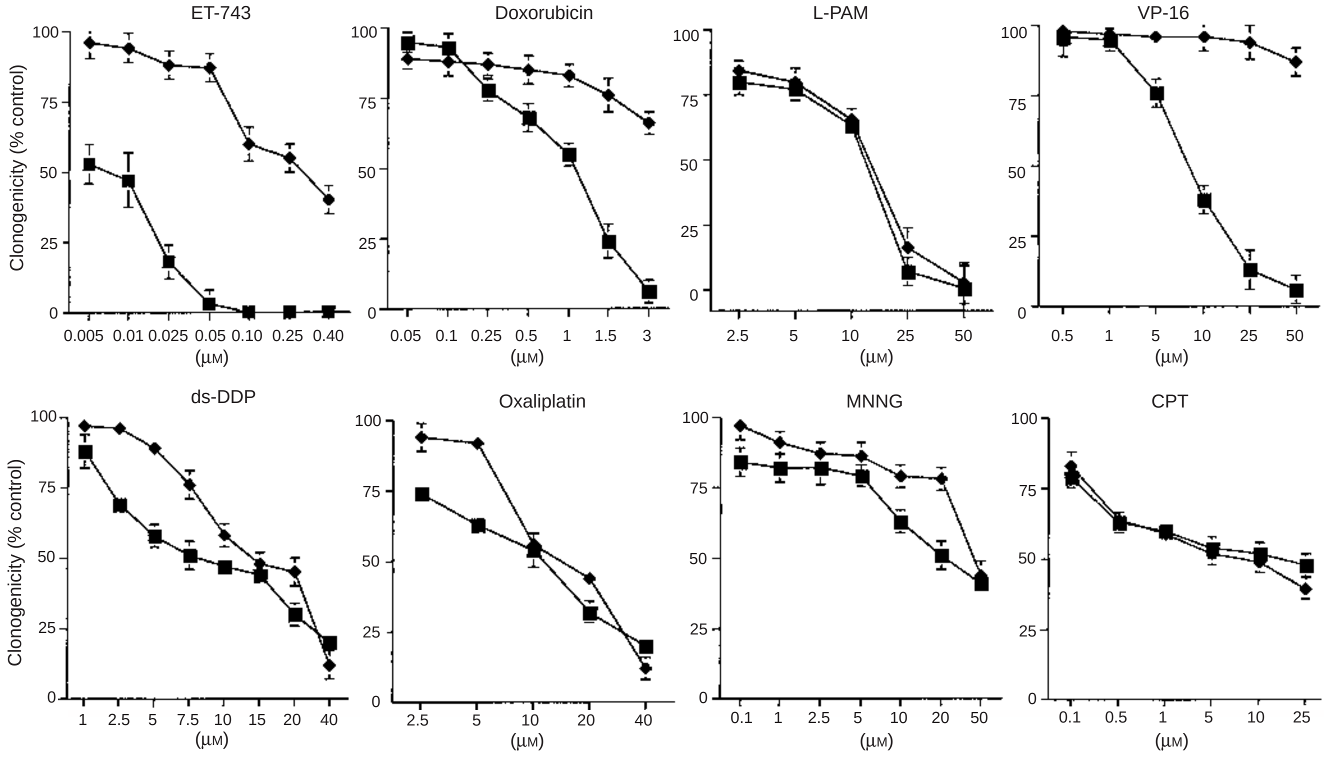

**Figure 6** Effect of ET-743, dox, L-PAM, VP-16, cis-DDP, Oxaliplatin, MNNG and CPT on the clonogenicity of Igrov-1 parental (■) and Igrov-1/25 ET (▲) cell lines. Each point is the mean of six independent replicates; bars represent s.d

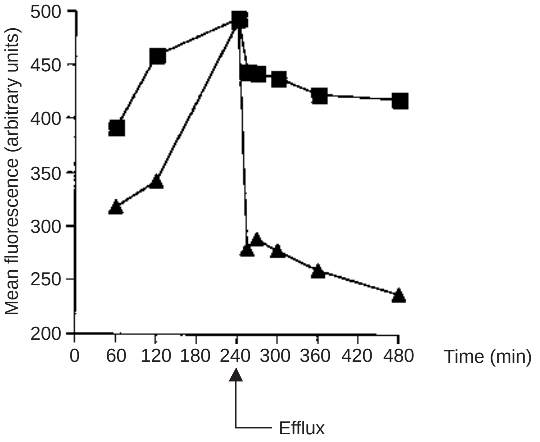

**Figure 7** Flow cytometric determination of doxorubicin uptake and efflux on Igrov-1/25 ET (▲) and Igrov-1 parental (■). Each point is the mean of three independent replicates. Standard deviation was less than 10%

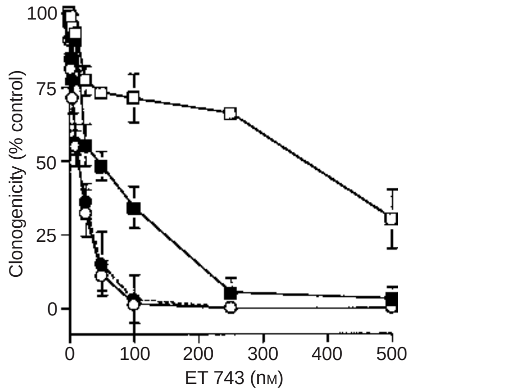

**Figure 8** Modulation of the clonogenic inhibitory effect of ET-743 by 1 μM PSC on Igrov-1 parental and Igrov-1/25 ET cell lines. Points are the mean of six independent replicates; bars represent s.d. (○) = Igrov-1 treated with ET-743 alone; (●) = Igrov-1 treated with ET-743 plus PSC; (□) = Igrov-1/25 ET treated with ET-743 alone; (■) = Igrov-1/25 ET treated with ET-743 plus PSC

tetraploid DNA (Figure 4). The resistance of Igrov-1/25 ET cell line to ET-743 was stable over 24 months in absence of ET-743.

### Expression of MRP and LRP

The expression of other proteins reported to be overexpressed in multidrug-resistant cells such as MRP and LRP has been investigated in Igrov-1/25 ET cell line in comparison with the parental Igrov-1 cell line. As shown in Figure 5 there is a small comparable expression of MRP and LRP in both Igrov-1/25 ET (Figure 5A) and Igrov-1 (Figure 5B) cell lines. The expression of P-gp was instead very marked in Igrov-1/25 ET cell line and undetectable in Igrov-1 cell line. In the same figure the results obtained in other cell lines used as positive control for MRP (i.e. A2780 cells, Figure 5C) or P-gp (i.e. Lovo/DX cells (Figure 5D) POGB/DX cells, Figure 5E) or MRP, LRP and Pgp (i.e. POGB/DX, Figure 5E) are also shown.

*© 2000 Cancer Research Campaign* 

### Cross-resistance pattern

As shown in Figure 6, a 50-fold increase in the ET-743 concentrations was required to cause a 50% reduction in the clonogenic potential of Igrov-1/25 ET cell line compared to sensitive Igrov-1 parental cell line. A comparable degree of cross-resistance was also found for dox and VP-16, while Igrov-1/25 ET cell line remained equally sensitive to L-PAM, MNNG and CPT. The sensitivity of Igrov-1/25 ET to cis-DDP and Oxaliplatin was slightly less than that of the parental cell line. This difference although small was statistically significant ($P < 0.01$).

### Dox uptake and efflux

Figure 7 shows the curves of uptake and efflux of dox in Igrov-1/25 ET and Igrov-1 cell lines. Dox uptake appeared to be slower in Igrov-1/25 ET than in Igrov-1 parental cell lines. At 4 h incubation the intracellular dox level reached similar values in both Igrov-1/25 ET and Igrov-1 cell lines. When dox was removed a very rapid drug efflux in the Igrov-1/25 ET was observed. At 480 min the mean fluorescence value was only 10% less than the maximal level reached at the end of treatment in Igrov-1 cell line, whereas it was about 50% less in Igrov-1/25 ET cell line.

### Reversal of resistance

PSC, a non-immunosuppressive cyclosporin derivative, is a potent agent used to reverse MDR associated with the overexpression of P-gp. In this study PSC was used at the subtoxic concentration of 1 μM in combination with ET-743. Figure 8 shows that PSC was able to reverse resistance to ET-743: in Igrov-1/25 ET cells the $IC_{50}$ values decreased from > 350 nM for ET-743 alone to < 50 nM for the PSC and ET-743 combination. No differences were observed in the Igrov-1 parental cell line when the clonogenic assay was performed with or without PSC.

### DISCUSSION

The present study shows that one of the mechanisms of resistance to the anticancer drug ET-743 is related to the overexpression of P-gp. Previous studies by this laboratory had shown that LoVo/DX cells resistant to dox because of the overexpression of P-gp were much less sensitive to ET-743 (Erba et al, 1996), indicating that ET-743 is a substrate for P-gp. The present study shows that both prolonged exposure with low ET-743 concentrations or short drug exposure with relatively high concentration results in the selection of resistant clones which overexpress mdr-1 gene. The clone that we have characterized in detail, from which we derived the cell line Igrov-1/25 ET, shows a typical behaviour of P-gp overexpressing cells. It is cross-resistant to dox and VP16, but not to alkylating agents, cis-DDP, Oxaliplatinum and CPT. We have not evaluated the intracellular concentration of ET-743 in Igrov-1 and Igrov-1/25 ET since the high potency of the drug would require an extremely sensitive analytical method or labelled compound, neither of which is available in our laboratory. The expression of P-gp in Igrov-1/25 ET cells suggested that the resistance is presumably related to a lower intracellular drug concentration. We have verified this by evaluating dox which could be easily estimated by determining the intracellular levels of fluorescence. These experiments indicated that dox efflux was much more efficient from Igrov-1/25 ET than from Igrov-1. It may be presumed that a similar change in the cellular pharmacokinetic of ET-743 is responsible for the resistance to this drug in Igrov-1/25 ET cells. This hypothesis is also supported by the fact that the cyclosporine analogue PSC, which is a potent mdr-1 reversing agent, could almost completely restore the sensitivity of Igrov/25 ET to ET-743 (Boesch et al, 1991).

Recently, Jin et al reported some preliminary data on the inhibition of the transcription of mdr-1 gene by ET-743 (Jin et al, 1999). The effect appeared due to the ability of ET-743 to inhibit the binding of the transcription factor NFY to a CCAAT box present in the mdr-1 gene promoter. Whereas these data confirm previous in vitro findings on the ability of ET-743 to prevent the formation of the complex of NFY with its consensus DNA sequence (Bonfanti et al, 1999) it is somehow surprising that by exposing cells with ET-743 we obtained resistant cell lines which overexpressed mdr-1 gene.

It may be hypothesized that we have selected cell lines which present alterations of the mechanism of regulation of the transcription of mdr-1 gene which might be not dependent upon NFY binding to the CCAAT box in the mdr-1 gene promoter. Future studies will be directed to verify this hypothesis. The fact that PSC can reverse the resistance to ET-743 can be of potential clinical interest since this cyclosporine analogue is currently being investigated with some other natural products which are also substrates of P-gp such as anthracyclines, epipodophyllotoxins or taxanes. It should be considered, however, that PSC might increase the concentration of ET-743 in some P-gp expressing normal tissues (e.g. liver), thus increasing its toxicity.

In order to evaluate the combination of ET-743 with reversing agents in vivo we have already transplanted Igrov-1/25 ET in nude mice and obtained a tumour which has maintained P-gp overexpression. This model, once characterized, will be potentially useful to identify new non-cross-resistant ET-743 analogue and to evaluate the efficacy of mdr-reversing agents to overcome ET-743 resistance.

### ACKNOWLEDGEMENTS

The generous contributions of the Italian Association for Cancer Research (AIRC) and of the Nerina and Mario Mattioli Foundation are gratefully acknowledged. We thank Dr Irene Floriani for the competent statistical advice.

### REFERENCES

Bénard J, Da Silva J, De Blois M, Boyer P, Duvillard P, Chiric E and Riou G (1985) Characterization of human ovarian adenocarcinoma line, IGROV 1, in tissue culture and in nude mice. *Cancer Res* **45**: 4970–4979

Binaschi M, Supino R, Gambetta RA, Giaccone G, Prosperi E, Capranico G and Zunino F (1995) MRP gene overexpression in a human doxorubicin-resistant SCLC cell line: alterations in cellular pharmacokinetics and in pattern of cross-resistance. *Int J Cancer* **62**: 84–89

Boesch D, Muller K, Pourtier-Manzanedo A and Loor F (1991) Restoration of daunomycin retention in multidrug-resistant P388 cells by submicromolar concentrations of SDZ PSC 833, a non-immunosuppressive cyclosporin derivative. *Exp Cell Res* **196**: 26–32

Bonfanti M, La Valle E, Fernandez Sousa Faro J-M, Faircloth G, Caretti G, Mantovani R and D'Incalci M (1999) Effect of ecteinascidin-743 (ET-743) on the interaction between DNA bindings proteins and DNA. *Anti-cancer Drug Des* **14**: 179–186

Broggini M, Grandi M, Ubezio P, Geroni C, Giuliani FC and D'Incalci M (1988) Intracellular doxorubicin concentrations and drug-induced DNA damage in a human colon adenocarcinoma cell line and in a drug-resistant subline. *Biochem Pharmacol* **37**: 4423–4431

 © 2000 Cancer Research Campaign

Cvitkovic E, Riofrio M, Goldwasser F, Delaloge S, Taamma A, Bijnen J, Jimeno JM, Mekranter B, Guzman C, Brain E and Misset JL (1999) Final results of phase 1 study of ecteinascidin-743 (ET-743) 24 hour (h) continuous infusion (CI) in advanced solid tumors (AST) patients (pts). *Proc ASCO* **18**: 180a

Erba E, Balconi G, Faretta M, Bergamaschi D, Guidi G, Jimeno J, Faircloth and D'Incalci M (1996) Cell cycle phases perturbations induced by new natural marine compounds. *Ann Oncol* **7**: 82

Ferrero M, Spyratos F, Le Doussal V, Desplaces A and Rouesse J (1990) Flow cytometric analysis of DNA content and keratins by using CK7, CK8, CK18, CK19, and KL1 monoclonal antibodies in benign and malignant human breast tumors. *Cytometry* **11**: 716–724

Ghielmini M, Colli E, Erba E, Bergamaschi D, Pampallona S, Jimeno J, Faircloth G and Sessa C (1998) In vitro schedule-dependency of myelotoxicity and cytotoxicity of Ecteinascidin 743 (ET-743). *Ann Oncol* **9**: 989–993

Guan Y, Sakai R, Rinehart KL and Wang AHJ (1993) Molecular and crystal structures of ecteinascidins: Potent antitumor compounds from the Caribbean tunicate *Ecteinascidia turbinata*. *J Biomol Struct Dyn* **10**: 793–818

Jimeno JM, Faircloth G, Cameron L, Meely K, Vega E, Gomez A, FernandezSousa-Faro JM and Rinehart K (1996) Progress in the acquisition of new marine-derived anticancer compounds: Development of ecteinascidin-743 (ET-743). *Drugs Fut* **21**: 1155–1165

Jin S, Lin Y, Magro PG and Scotto KW (1999) Repression of MDR1 gene transcription by ecteinascidin 743. *Proc Am Ass Cancer Res* **40**: 314

Krishan A, Sauerteig A, Andritsch I and Wellham L (1997) Flow cytometric analysis of the multiple drug resistance phenotype. *Leukemia* **11**: 1138–1146

Limonta M, Ubezio P, Catapano CV, Conter V, Costato C, Specchia G, Liso V, Barbui T, Giudici G and D'Incalci M (1991) Doxorubicin and m-AMSA induced DNA damage in blast cells from AML patients. *Leukemia Res* **15**: 19–24

Moore II, BM, Seaman FC and Hurley LH (1997) NMR-based model of an ecteinascidin 743-DNA adduct. *J Am Chem Soc* **119**: 5475–5476

Pommier Y, Kohlhagen G, Bailly C, Waring M, Mazumder A and Kohn KW (1996) DNA sequence- and structure-selective alkylation of guanine N2 in the DNA minor groove by ecteinascidin 743, a potent antitumor compound from the Caribbean tunicate *Ecteinascidia turbinata*. *Biochemistry* **35**: 13303–13309

Rayan DP, Supko JG, Eder JP, Lu H, Chabner B, Roper K, Baccala P, Bonefant J, Faircloth G, Guzman C, Jimeno J and Clark JW (1999) A phase I and pharmacokinetic trial of ecteinacidin-743 (ET-743) administered as a 72 hour continuous infusion. *Proc ASCO* **18**: 188a

Rinehart KL, Holt TG, Fregeau NL, Stroh JG, Keifer PA, Li LH and Martin DG (1990) Ecteinascidins 729, 743, 745, 759A, 759B, and 770: Potent antitumor agents from the Caribbean tunicate *Ecteinascidia turbinata*. *J Org Chem* **55**: 4512–4515

Sambrook J, Fritsch EF and Maniatis T (1989) *Molecular Cloning*. Cold Spring Harbor Laboratory Press, Cold Spring Harbor

Valoti G, Nicoletti MI, Pellegrino A, Jimeno J, Hendriks H, D'Incalci M, Faircloth G and Giavazzi R (1998) Ecteinascidin-743, a new marine natural product with potent antitumor activity on human ovarian carcinoma xenografts. *Clin Cancer Res* **4**: 1977–1983

Zewail-Foote M and Hurley LH (1999) Ecteinascidin 743: a minor groove alkylator that bends DNA toward the major groove. *J Med Chem* **42**: 2493–2497

*© 2000 Cancer Research Campaign*
